# Resuscitative thoracotomies and open chest cardiac compressions in non-traumatic cardiac arrest

**DOI:** 10.1186/1749-7922-9-54

**Published:** 2014-10-20

**Authors:** Daniel Kristoffer Kornhall, Thomas Dolven

**Affiliations:** Department of Anaesthesiology, University Hospital of North Norway, Sykehusveien 38, Tromsoe, 9038 Norway; Department of Anaesthesiology, Haukeland University Hospital, Bergen, Norway

**Keywords:** Resuscitation, Cardiac arrest, Open chest cardiac compressions, Thoracotomy, Non-traumatic, CPR

## Abstract

Since the popularisation of closed chest cardiac compressions in the 1960s, open chest compressions in non-traumatic cardiac arrest have become a largely forgotten art. Today, open chest compressions are only rarely performed outside operating theatres. Early defibrillation and high quality closed chest compressions is the dominating gold standard for the layman on the street as well as for the resuscitation specialist. In this paper we argue that the concept of open chest direct cardiac compressions in non-traumatic cardiac arrest should be revisited and that it might be due for a revival. Numerous studies demonstrate how open chest cardiac compressions are superior to closed chest compressions in regards to physiological parameters and outcomes. Thus, by incorporating resuscitative thoracotomies and open chest compressions in our algorithms for non-traumatic cardiac arrest we may improve outcomes.

## Review

### Introduction

Outcomes in out-of-hospital non-traumatic cardiac arrest have displayed marginal improvement since many decades. With a few notable exceptions, survival to discharge rates are less than 10% [1]. Thus, it is exciting to see how future concepts in non-traumatic cardiac arrest management are constantly being developed and evaluated. One concept, though certainly not a new one, that frequently resurfaces in cardiac arrest literature is open chest cardiac massage [2, 3]. Open chest cardiopulmonary resuscitation (OC-CPR) is the direct massage of the heart as opposed to the conventional closed chest cardiac compressions (CC-CPR). Numerous human and animal studies demonstrate how OC-CPR results in significantly improved hemodynamics and outcomes when compared to CC-CPR. Despite that, OC-CPR has not since the 1960s been considered a mainstream resuscitative intervention outside cardiac surgery and outside operating theatres.

This is partly explained by how the resuscitative thoracotomy, that allows access to the arrested heart, requires an operating theatre and controlled surgical conditions as it is an massively invasive intervention. Such dogma will inevitably delay initiating OC-CPR so that it’s benefits are lost. Animal studies indicate the benefits of OC-CPR are lost when direct cardiac massage is initiated after more than 20 minutes of cardiac arrest [4]. Instead, CC-CPR has become the dominant intervention in non-traumatic cardiac arrest since it was popularised in the 1960s.

In contrast, resuscitative thoracotomies with OC-CPR are frequently performed in emergency departments in *traumatic* cardiac arrest caused by thoracic injuries. Importantly, since about a decade, resuscitative thoracotomies are even performed outside hospitals. Air ambulance services have trained their clinicians to successfully perform thoracotomies in the prehospital setting for treating traumatic cardiac arrest [5].

In light of that, perhaps it is time for thoracotomies and direct cardiac massage to refind their place in the resuscitation of non-traumatic cardiac arrest. By performing OC-CPR at a much earlier stage, in the emergency departments or even outside our hospitals, we could improve outcomes.

Open chest heart massage is not a novel concept. As a mainstream concept OC-CPR predates our gold standard CC-CPR by almost half a century. The method was first described on laboratory animals by professor Moritz Schiff in 1874. In 1901 norwegian physician Kristian Igelsrud performed the first successful resuscitation from cardiac arrest in a human subject using open chest compressions when a patient arrested during an elective hysterectomy [6]. From then on, OC-CPR would for half a century remain the dominant method for cardiopulmonary resuscitation. Several large case series were published during that era proving the methods efficacy. In 1953 Stephenson et al. published a data from 1200 theatre cardiac arrests that had open chest CPR. The recovery rate was 28% [7]. In 1954 Briggs et al. reported theatre cardiac arrest from the Massachusetts General Hospital during a 30 year period. In the patients where open chest CPR was initiated within 4 minutes, 58% recovered and were neurologically intact [8]. To some extent this data can be compared to the Beth Israel outcomes study published in 1983. The authors, Bedell et al., reported survival rates of only 14% in patients who suffered in-hospital cardiac arrest who had closed chest compressions [9].

Despite these promising findings, open chest compressions was about to be marginalised from the mainstream when, in the 1960s, Kouwenhoven et al. popularised closed chest compressions. In fairness, CC-CPR was described already in 1786 by Enfield surgeon John Sherwin, but it would remain an obscure method until the 1960s when closed chest compressions would become the gold standard for the layman on the street as well as for the resuscitation specialist. Since then, open chest compressions in non-traumatic cardiac arrest is rarely performed outside operating theatres or on postoperative care patients who recently had thoracotomies for cardiac surgery.

## Methods

This review intends to explore the possible benefit of OC-CPR over CC-CPR in non-traumatic cardiac arrest. We also intend to describe how OC-CPR could be implemented into hospital or prehospital cardiac arrest management. We aimed to identify studies that directly compare OC-CPR with CC-CPR in regards to physiological variables and outcomes when implemented after non-traumatic cardiac arrest. Studies were identified by searching Medline and Embase. An exhaustive manual search of citations in relevant reviews and studies was also performed. The sections on the history of OC-CPR, the physiology of CPR and on the practical aspects of performing a resuscitative thoracotomy were based on an non-systematic review of current literature and on the authors’ experiences from working in prehospital emergency medicine.

## Results

### Evidence for OC-CPR providing improved physiology over CC-CPR

We know that early defibrillation and quality cardiac compressions are what matters most for achieving return of spontaneous circulation (ROSC) in non-traumatic cardiac arrest. The aim of cardiac compressions is to artificially produce cardiac output and coronary perfusion pressures. The higher the artificial coronary perfusion pressure we achieve the more likely we are to achieve ROSC. While achieving a high coronary perfusion pressure certainly does not guarantee ROSC, we know that failure to perform effective compressions inevitably result in failure to resuscitate. This was convincingly demonstrated in 1990 by Paradis *et al.* as they compared coronary perfusion pressures generated by CC-CPR with and without return of spontaneous circulation. The authors could demonstrate how CC-CPR generating less than 15 mmHg was associated with 100% failure to resuscitate [10]. With CC-CPR we rarely achieve that outside the lab. Several studies report abysmally low mean coronary perfusion pressures, often in the range of 1–9 mmHg [11, 12].

Here is where OC-CPR might refind it’s place in resuscitation of non-traumatic cardiac arrest. Open chest compressions are significantly more efficient and generate a significantly greater coronary perfusion pressure gradient. Numerous animal studies demonstrate improved aortic and coronary perfusion pressures with OC-CPR over CC-CPR [13–15]. Typically coronary perfusion pressures more than double. In 1988, Raessler and Kern demonstrated in a canine experiment how coronary perfusion pressure with OC-CPR was 64 mmHg compared to only 21 mmHg with CC-CPR [16]. In a 2003 animal study, Benson et al. obtained coronary perfusion pressures averaging 38,2 with OC-CPR compared to 20,3 mmHg with CC-CPR [17]. Cerebral perfusion also seems to improve with OC-CPR. In a study measuring cerebral blood flow (CBF), Byrne et al. could demonstrate near-normal CBF with OC-CPR compared to 30% with CC-CPR [18]. Similar findings have been confirmed by other studies [19].

The few human studies that report haemodynamic parameters confirm the findings from animal studies. In 1995 Boczar et al. demonstrated, in human non-traumatic cardiac arrest, how average coronary perfusion pressures increased from 7.3 +/- 5.7 mmHg with closed chest compressions to 32.6 +/- 17.8 mmHg after performing a left lateral thoracotomy and continuing with open chest direct cardiac compressions [20]. In 1965 Del Guercio et al. demonstrated how open-chest compressions resulted in a mean cardiac index of 1.31 L/min/m2 compared to only 0.61 L/min/m2 during closed-chest cardiac massage [21].

Thus, there is ample evidence for haemodynamic parameters improving with OC-CPR when compared to CC-CPR. With OC-CPR we can generate higher coronary perfusion pressures and cardiac output. This should translate into improved outcomes. With OC-CPR we should be able to increase ROSC-rates and reduce multi-organ ischaemic injury.

### Evidence of improved outcomes with open chest CPR

While there are few human outcome studies, numerous animal studies of good quality have been published. These consistently demonstrate the superiority of OC-CPR [22]. The likelihood of achieving ROSC is significantly increased.

In 1984, Sanders et al. randomised dogs into receiving either OC-CPR or CC-CPR after 15 minutes of unsuccessful standard CC-CPR. In the OC-CPR group, four out of five dogs survived compared to none in the control group who had closed chest cardiac compressions throughout [13]. In 1987 Kern et al. performed a similar study demonstrating how all (n =14) animals were successfully resuscitated with OC-CPR initiated after 15 minutes of failed CC-CPR. In the control group, who had CC-CPR throughout, only 5 out of 14 had return of spontaneous circulation. The authors also reported favourable 7 day survival in the group who had OC-CPR [14]. In 2005, Benson et al. induced cardiac arrest in dogs then randomised them into receiving either closed chest CPR or open chest CPR after five minutes of no intervention. All dogs who had OC-CPR were resuscitated within 15 minutes. All of them had neurologically favourable outcomes after three days. In contrast, only three out of seven dogs who received CC-CPRs were alive at three days. All three suffered severe neurologic deficits [17].

There are fewer human studies to rely on. As of today only five studies exist. Only two detail outcomes in out-of-hospital cardiac arrest (OOHCA) while the remaining describe open chest cardiac compressions in association with cardiac surgery.

In 1993, Takino and Okada published a cohort study of 93 patients who were delivered to their emergency department after suffering non-traumatic OOHCA. 26 of these patients had OC-CPR through left lateral thoracotomies. The remaining 69 patients had conventional cardiopulmonary resuscitation. In the open chest cardiac compressions group, ROSC was achieved in 15 patients (58%) In the standard closed chest cardiac compression group 30% of patients achieved ROSC [23]. In their 1995 paper, Boczar et al. studied 10 patients who were delivered to an emergency department in cardiac arrest after suffering a witnessed cardiac arrest. After 5 minutes of unsuccessful conventional CPR a left lateral thoracotomy was performed. The authors noted how mean coronary perfusion pressure rose from 7,3 to 32 mmHg and three of the ten ‘unsalvageable’ patients obtained return of spontaneous circulation.

While the results from the two existing OOHCA arrest studies are striking they suffer from drawbacks. In both studies open chest cardiac compressions are instituted at a very late stage. In the Takino paper the time from emergency dispatch phone call to ED admission was 19 minutes. Then it took another 10,5 minutes to establish open chest cardiac compressions. In the Boczar study, the patients are deemed unsalvageable before being included in a study that primarily aimed to measure coronary perfusion pressure.

The remaining studies describe outcome data from postoperative patients who suffer cardiac arrest after having had cardiac surgery. They provide some insight as to what can be expected when OC-CPR is initiated at a much earlier stage.

Anthi et al. describe 29 patients who suffer cardiac arrest within 24 h of having cardiac surgery. All patients initially had 3 to 5 minutes of conventional CPR before resternotomy and open chest cardiac compressions were performed. Of the 16 patients who required open chest cardiac compressions, 14 were successfully resuscitated [24]. A similar retrospective review by Pottle et al. demonstrated successful resuscitation in 46% of cardiac surgery patients who suffered postoperative cardiac arrest [25]. In 2011 Karhunen et al. described 76 patients who suffered cardiac arrest following coronary artery bypass grafting. After immediate resternotomy and OC-CPR there were 62(82%) survivors [26].

## Discussion

Thus, there is ample outcome evidence from animal studies proving OC-CPR is superior to CC-CPR. Data from the few existing human studies report OC-CPR outcomes that are significantly better than outcomes where CC-CPR is the default intervention. The human studies, however, must be very carefully interpreted. They suffer from the intervention being performed far too late or from describing patients who recently had cardiac surgery. The latter group are exposed to a unique set of patophysiologic mechanisms causing them to suffer cardiac arrest. Still, if we were we to find OC-CPR a viable way forward in managing non-traumatic cardiac arrest in a general population, many questions would need to be addressed. Amongst other issues, we need to address how would we implement it into our existing cardiac arrest algorithms. We also need to consider the risks and complications of introducing resuscitative thoracotomies in cardiac arrest.

### Resuscitative thoracotomy and open chest compressions in practice

If we were to introduce OC-CPR in non-traumatic cardiac arrest we need to implement a thoracotomy and direct cardiac massage into existing algorithms. Guidelines emphasise early recognition, early bystander CPR, early chest compression with minimised interruptions, early defibrillation and optimal post-ROSC care [27]. With OC-CPR, the same performance goals apply. As with conventional CPR, in OC-CPR cardiac massage must be initiated as early as possible. In a canine model, Sanders et al. demonstrated how 75% of animals were resuscitated when OC-CPR was initiated within 15 minutes. If thoracotomy and OC-CPR was delayed to 20 minutes ROSC-rates dropped to 40% [28].

I order to secure early access to the heart and initiate OC-CPR, thoracostomies must be performed in the emergency department or even as a prehospital intervention. These resuscitative thoracotomies with OC-CPR are already, since the 1960s, being performed in emergency departments and operating theatres on patients suffering cardiac arrest after thoracic trauma [29, 30]. Since more than a decade, resuscitative thoracotomies are even performed outside hospitals by prehospital clinicians. Prehospital clamshell thoracotomies have successfully been integrated into algorithms for traumatic cardiac arrest [5].

Access to the heart and OC-CPR must be achieved within a minutes. While several incisions have been used for performing resuscitative thoracotomies, the left anterior thoracotomy (LAT) is typically considered the standard method as it provides rapid access to the heart and descending aorta [31]. The bilateral anterior thoracotomy (Clamshell) has been demonstrated to provide better access to thoracic structures in thoracic trauma but for the purposes of OC-CPR in non-traumatic cardiac arrest the LAT could suffice [32].

LAT is performed on the supine patient. After a rapid skin preparation with anti-septic agent, an scalpel incision is made in the 5th intercostal space, extending from the mid-axillary line to the border of the sternum. The incision is made through skin and subcutaneous tissues down to the intercostal muscles. The intercostal muscles can then be cut using a combination of scalpel, trauma scissors and blunt dissection while taking care not to lacerate the lung. Access to the thoracic cavity is achieved by opening the thorax with a rib spreader or similar instrument. Cardiac massage is then performed by reaching into the thorax with the wrists together at the apex and the hands reaching around the heart and pericardium. With a rhythmic bellow-like motion the heart is compressed [33]. OC-CPR is then performed in accordance with conventional CC-CPR algorithms. Changes in cardiac activity during resuscitation will be directly observed, felt by the clinician performing cardiac massage or will be detected during conventional algorithm rhythm and pulse checks. Ventricular fibrillation could be managed with internal defibrillator paddles, if available, or by external defibrillation [34]. ROSC would be identified either during a normal algorithm pulse check or as palpable organised ventricular action.

After achieving ROSC the patient will be subjected to the normal treatment pathways after cardiac arrest. About 70% of cardiac arrests are caused by coronary heart disease and the majority of patients would travel down a treatment pathway with coronary ischaemia in mind [35]. Current guidelines recommend immediate referral of these patients to a facility capable of coronary angiography and cardiac catheterisation unless there is an obvious non-cardiac cause when a local hospital with intensive care capability is sufficient [27]. Definitive treatment of coronary occlusion include balloon angioplasty, implantation of stents, thrombectomy or intracoronary administration of low-dose thrombolysis. Alternatively, occluded coronary vessels are managed with open chest surgery and coronary artery bypass grafting [36]. Besides standard intensive care, post-ROSC care in patients with cardiac arrest of a cardiac origin could require the use of numerous treatment modalities and supportive measures depending on the clinical situation. Cardiopulmonary by-pass, intra-aortic ballon pumps, surgery as well as therapeutic hypothermia are all common treatment options. The presence of a resuscitative thoracotomy incision will not significantly interfere with the majority of these interventions nor will it interfere with standard intensive care other than requiring post-surgical care in regards to the thoracotomy incision. Still, the addition of thoracotomy and OC-CPR to conventional cardiac arrest management will introduce a particular set of risks and complications.

### Complications associated with thoracostomies and open chest

Implementing OC-CPR in non-traumatic cardiac arrest would introduce the complications associated with thoracotomies. A resuscitative thoracotomy is a dangerous procedure, requiring sharp instruments, that is by it’s nature performed under uncontrolled conditions. Numerous procedural complications exist. An improperly placed incision could restrict access to the heart. Performing a resuscitative thoracotomy can result in injury to nerves, blood vessels, lungs and heart. Phrenic, intercostal and intercostal nerves risk being lacerated or cut. A careless or unfortunate incision could result in pulmonary injury. Cardiac complications in the form of injury to the pericardium, myocardium and coronary vessels could be the result of the incision or from cardiac massage. Finally, bleeding from intercostal and pulmonary vessels or the internal mammary artery must be anticipated and controlled [37]. Bleeding from the incised tissues would always be a complicating issue. Bleeding is to some extent restricted as post-ROSC patients, as part of the post-cardiac arrest syndrome, often are in a state of low cardiac output and low blood pressure [38]. In any case tissue bleeding needs to be addressed as soon as appropriate. Hemostasis could be achieved through direct pressure, dressings, surgery, electrocoagulation or through the use of hemostatic agents [39]. Bleeding would be aggravated in patients treated with prophylactic anticoagulation. Still, anticoagulation does not present an absolute contraindication to neither resuscitative thoracotomies nor OC-CPR given that they are potentially life-saving interventions.

Special consideration must be given to patients who require thrombolysis after ROSC. Massive pulmonary embolism and brain stroke are examples of diseases that are associated with non-traumatic cardiac arrest and are sometimes treated with thrombolysis. In these cases the thoracotomy incision would present a relative contraindication to thrombolysis, favouring other treatment options such as embolectomy and local thrombolysis.

## Conclusion

Numerous animal studies demonstrate the superiority of OC-CPR over CC-CPR with improved physiologic parameters, improved chances of achieving ROSC and improved outcomes. The few human studies that exist are hard to draw conclusions from but seem to confirm conclusions from the animal studies. Implementing OC-CPR in existing cardiac arrest algorithms requires the implementation of resuscitative thoracotomies in non-traumatic cardiac arrest. While this is controversial, one needs to consider that resuscitative thoracotomies are already being performed globally as an intervention against traumatic cardiac arrest in emergency departments. Since about a decade, resuscitative thoracotomies are even performed in the pre-hospital setting by specially trained physicians. Incorporating OC-CPR into our arsenal would, however, present many new challenges. Clinicians would require training in a highly invasive procedure with numerous, potentially serious, complications. Complications are mostly associated with the resuscitative thoracotomy and include damage to nerves, blood vessels, lung and the heart. It would also invariably result in bleeding from the incised tissues that would need to be controlled. Still, if improved outcomes outweigh the impact of complications then OC-CPR could be a way forward in the management of non-traumatic cardiac arrest where outcomes remain poor despite decades of research and innovation.
